# Research on the Effect of Narcissistic Leadership on Employee Job Embeddedness

**DOI:** 10.3389/fpsyg.2022.927529

**Published:** 2022-07-07

**Authors:** Heli Wang, Runkai Jiao, Feifei Li

**Affiliations:** School of Psychology, Northeast Normal University, Changchun, China

**Keywords:** narcissistic leadership, leader-member exchange (LMX), perceived insider status, job embeddedness, chain mediation

## Abstract

Narcissistic leadership is the synthesis of narcissistic personality traits and leadership behaviors that are motivated mainly by self-interest needs and arrogant beliefs. Such leadership style has multiple effects on organizations and employees. The amplifying influence of narcissistic leadership on their subordinates has become a hot topic in the field of organizational behavior. Based on the social exchange theory and the resource conservation theory, the current study constructs a chain mediation model of narcissistic leadership affecting employees’ job embeddedness with 405 corporate employees as survey respondents. The results of data analysis show that narcissistic leadership is significantly and negatively related to employees’ job embeddedness; Leader-member exchange (LMX) and perceived insider status not only play a mediating role between narcissistic leadership and job embeddedness but also play a chain mediating roles in the relationship between narcissistic leadership and job embeddedness. Our findings deepen the theoretical exploration of narcissistic leadership and help all types of organizations to improve their leadership practices.

## Introduction

As more and more new generation of the post-90s and post-95s employees enter the workplace, they bring many fresh elements of the times to the enterprises. The growth of this group is accompanied by the characteristics of the times with the rapid development of electronic information equipment and Internet technology. Therefore, it reflects the characteristic of personality, behavior, and psychology that are completely different from those of previous generations. The new generation employees are bold, open, and unique; they pursue personal values and emphasize respectfulness; they are more emotional and expressive; they face a more competitive environment and prefer new things. With the advent of the new era, this group is increasingly becoming an emerging source and active factor in all industries, and is the main force in the talent market faced by enterprises and organizations. However, the contradiction is that most of the leaders in Chinese organizations have different thinking patterns and principles from young employees due to intergenerational differences, and along with many problems such as work task pressure and salary, new generation employees’ sense of attachment, loyalty, and embeddedness to the organization are getting lower and lower, which has seriously hindered the improvement of economic efficiency and sustainable development of enterprises (Xu et al., 2021; Yang et al., 2021). The main problem that enterprise managers are facing now is how to take appropriate measures to guide, encourage and retain new generation employees. In this context, clarifying the influencing factors of new generation employees’ job embeddedness and reducing their turnover rate have become a hot issue for enterprises to solve (Fang et al., 2020).

Job embeddedness, a measurement variable of employee turnover, has received extensive academic attention (Mitchell et al., 2001; Awan et al., 2021; Elshaer and Azazz, 2021). Job embeddedness reflects the relationship between the individual and the organization, especially the degree of trust and dependence of the individual on the organization (Liao et al., 2021). A review of previous literature reveals that scholars have mainly studied the influencing factors of job embeddedness from the following aspects: individual factors (e.g., age, gender, and education), organizational factors (e.g., job rotation, socialization strategies, and employment relationships) (Cepale et al., 2021; Perinelli et al., 2022), and environmental factors (e.g., talent policies and human environment perceptions) (Zhang et al., 2012; William et al., 2014; Ghosh and Gurunathan, 2015). However, it is worth noting that there are few studies on the impact of leadership style and traits on employees’ job embeddedness. “Narcissism,” a common psychological and stylistic trait of senior managers, has been shown to have an impact on subordinates’ psychology and further provoke their negative attitudes and behaviors, such as cynicism, low job satisfaction, silent behavior, and turnover intentions (Alhasnawi and Abbas, 2021). Existing research has also proved that narcissistic leadership is more likely to occur in the culture with a high tendency toward collectivism (Weng et al., 2018; Xiao et al., 2018). Accordingly, the present study constructs a theoretical model of narcissistic leadership influencing employees’ job embeddedness and analyzes the mediating effect of LMX and perceived insider status based on the Chinese context, which not only enriches the relevant empirical research related to narcissistic leadership and employees’ job embeddedness, but also provides a theoretical reference and practical guidance for organizations to mitigate the dark side of narcissistic leadership and stimulate employees’ positive work attitudes and behaviors.

## Theoretical Background and Hypotheses

### New Generation Employee

The concept of the new generation originates from the research of Egri and Ralston (2004), who classified three generations in the Chinese workplace as the construction generation, the cultural revolution generation, and the new generation, while other scholars defined employees born between 1980 and 2000 as the generation Y employees. Most scholars define the new generation as the post-90s. The present study suggests that the new generation refers to the group born after 1990, aged between 18 and 32, who have already worked. They start to take more responsibilities and gradually become the backbone of the workplace.

The new generation employees have distinctive group characteristics. (1) They are bold and open, interfere with the shackles of old-fashioned thinking, dare to challenge authority and pursue equality. In dealing with things, the new generation employees show more bold and innovative, and their thinking is more active. (2) They pursue personal values and emphasize respectfulness. Most of the new generation employees are only children, with favorable family conditions and easy upbringing, strong sense of personal consultation and autonomy, focus on self-perception, and pursue relatively free working environment and work experience. (3) Their behavior is susceptible to emotional influences. The growth process of the new generation employees is synchronized with the rise of China’s Internet industry. They have mastered the use of various new Internet technologies and tools, acquired a large amount of information and knowledge, and received multiple value orientations. At the same time, they also become more impetuous and emotional. (4) They are the generation that has fully received 9-year compulsory education and coincided with the expansion of higher education. Their overall education level is significantly higher than other generations. Therefore, the overall characteristics of the new generation employees are “three highs and one low,” i.e., high education level, high career expectations, high material and spiritual enjoyment requirements, and low work tolerance (Fang et al., 2020; Xu et al., 2020, 2021).

The new generation employees in the workplace also have unavoidable negative characteristics. They are too self-centered and will magnify their personal feelings, which is not conducive to teamwork. Their value orientation is pragmatic and they lack of dedication. They seldom encounter frustration and failure in the growth process, poor stress resistance in the actual work, many career options, low organizational loyalty.

### The Related Research on Narcissistic Leadership

Narcissism was first introduced into the field of psychiatry in the late nineteenth century by psychiatrist Ellis, who defined narcissism as a tendency to lose one’s sexuality and become almost completely absorbed in self-appreciation. In 1968, the American Psychiatric Association proposed that narcissim is a pathological phenomenon in which psychological interest focuses on itself. In 1980, the third edition of the American Diagnostic Manual of Mental Disorders (DSM-III) set specific clinical criteria for narcissistic personality, including arrogance, desire for success and power, and lack of empathy.

In the 1920s, psychologist Freud proposed the view that most leaders have certain narcissistic tendencies. Subsequently, many scholars began to study narcissistic leadership as a specific leadership style. Modern research confirms Freud’s view from the other side that narcissistic people tend to be perceived as smarter than the average person and therefore more likely to be leaders in teams. In the 1980s, De Vries and Miller (1985) discussed the relationship between leaders’ effectiveness and dysfunction and their narcissistic personality traits, suggesting that leaders’ behaviors are influenced by their degree of narcissism. Rosenthal and Pittinsky (2006) first put forward the concept of “narcissistic leadership,” arguing that narcissistic leaders are motivated by personal needs and perceptions, and that for them, personal interests outweigh organizational interests. According to Chinese scholars Huang and Li (2014), narcissistic leaders often appear to be charismatic, have superior vision, are strongly egoistic and seek superiority, are blindly arrogant and enjoy widespread attention, have an extremely strong aversion to criticism, and reject negative feedback. Rosenthal and Pittinsky (2006) and Grijalva and Harms (2014) suggested that narcissistic leaders are characterized by grandiosity, power-seeking, and a strong desire for success. Khoo and Burch (2008) argued that in general, the traits of narcissistic leaders and their performance at work have more negative than positive effects.

Throughout the research on the common understanding of the connotation of narcissistic leadership, it can be found that narcissistic leadership is a combination of narcissistic personality traits and leadership behaviors, such leaders have a strong sense of self and self-interest motivation, but at the same time, they desire the recognition and praise of others to maintain their inflated self-perceptions and show fragile self-esteem (Duchon and Burns, 2008; Ouimet, 2010), so they can be described as “arrogant and fragile”.

### Narcissistic Leadership and Employee Job Embeddedness

Rosenthal and Pittinsky (2006) were the first to point out that leaders are narcissistic leaders when their behaviors are motivated primarily by extremely selfish personal needs and are not driven by the interests of the organization they lead. Narcissistic leaders are usually arrogant, irritable, capricious, stubborn, and have a significant impact on employees’ attitudes and behaviors (Ouimet, 2010). Job embeddedness refers to the comprehensive factors that induce individuals to stay in the organization, and the core content contains three points: connection, matching and sacrifice. Connection refers to the degree of interaction between employees and colleagues or leaders in the work process; matching refers to the degree of adaptation or integration of employees into their work environment; and sacrifice refers to the opportunity cost of material or spiritual benefits that may be lost by leaving the organization (Mitchell et al., 2001; Lee et al., 2004; William et al., 2014). Job embeddedness is distinctly different from similar concepts in organizational behavior and sociology. Job embeddedness encompasses factors outside the workplace, including evaluation based on emotion and cognition. It is not inherently affective, nor is it limited to an individual’s identification with or attachment to the organization. Job satisfaction is closely related to the workplace and is an immediate attitudinal variable. Organizational commitment is also closely related to the workplace, which emphasizes continuous efforts for the organization and reflects employees’ strong desire for organizational membership. The difference between job embeddedness and job satisfaction and organizational commitment is that job embeddedness includes both organizational and community factors. Thus it is broader in scope than job satisfaction and organizational commitment. In addition, job embeddedness is a variable used to evaluate the extent to which individuals are attached to the organization, rather than focusing on why individuals are attached to the job and whether they are willing to adopt such an attachment (Yuan and Chen, 2008).

It has been shown that leader styles and traits are significantly related to the degree of job embeddedness of subordinates (Khorakian et al., 2021; Amankwaa et al., 2022). Therefore, based on the social exchange theory and the resource conservation theory, the present study hypothesizes that narcissistic leadership has a significant negative impact on employees’ job embeddedness for the following reasons. On the one hand, social exchange theory states that when employees receive moral encouragement and material help from their leaders, they will reward the leader and the organization by putting in work or supporting the leaders and a series of beneficial activities for the organization out of reciprocity (Cropanzano et al., 2017; Wang Z. et al., 2021). Conversely, when leaders are arrogant, sensitive, angry, or lacking in empathy toward employees, employees perceive that the exchange relationship is unbalanced or that they are being used, resulting in behavioral outcomes that are detrimental to the organization, such as increased emotional exhaustion and reduced organizational commitment and embeddedness (Glad, 2002). Thus, according to social exchange theory, the capricious and oversensitive characteristics of narcissistic leaders will undermine the job embeddedness of new generation employees. Employees view leaders as agents of the organization and attribute to the organization the way leaders evaluate and treat them. The capricious nature of narcissistic leaders makes them prone to conduct negative management behaviors in order to maintain their own interests and authority, which deteriorates the perception of good relationship between employees and the organization and leads to negative responses from employees that reduce their job embeddedness (Ogunfowora, 2013; Haggard and Park, 2018; Mackey et al., 2018). At the same time, the hypersensitivity of narcissistic leaders leads them to be suspicious and self-defensive and lack trust in their subordinates (Rosenthal and Pittinsky, 2006), which can severely weaken employees’ perceptions of leadership support and reduce their job embeddedness. On the other hand, resource conservation theory, as an explanation of how individuals in an organization handle resources in the face of stress, reflects the characteristics of individuals’ pursuit of new resources and maintenance of existing resources. Employees are always trying to actively acquire new resources and avoid the loss of existing resources in order to achieve success. According to resource conservation theory, abusive leadership depletes employees of the self-resources needed to maintain appropriate behaviors, such as attention, willpower, and self-esteem (Hobfoll, 1989; Tepper et al., 2007). When the negative traits of narcissistic leadership are on display, individuals will seek new resources in response to this pressure. However, traits such as narcissistic leadership egoism attribute organizational resources and success to themselves. These pressures disrupt the balance of individuals’ resources, making it significantly more difficult to acquire new resources and maintain existing resources from loss (Saks and Gruman, 2018). As a result, employees will eventually choose to reduce their job embeddedness or even leave the organization in order to preserve and protect their resources from losses. Thus, after being subjected to hostility, public denigration and threats from narcissistic leaders, employees’ emotional and respectful needs and other self-resources are greatly depleted, which directly manifests itself in the form of alienation from the organization and reduced inner connection and integration with the organization. Therefore, we hypothesize the following:

**Hypothesis 1.** Narcissistic leadership is negatively related to employees’ job embeddedness.

### The Mediating Role of Leader-Member Exchange

LMX refers to the superior-subordinate relationship formed through contact and communication between the superior leaders and the immediate subordinates in the organization (Martin et al., 2018; Andersen et al., 2020). Due to the limitation of time, energy and resources, it is impossible for leaders or managers to establish equal interaction with each employee. They always divide their subordinates into “insiders” and “outsiders” intentionally or unintentionally according to some unwritten criteria (Chen et al., 2009; Lam et al., 2015). Leaders’ care, understanding and support for employees can effectively improve LMX. On the contrary, if leaders do not respect employees and ignore their needs and development, a low-quality of LMX will be established between leaders and employees or subordinates (Tang et al., 2020). Narcissistic leaders tend to be self-centered in the process of working with their subordinates, focusing only on expressing and implementing their own ideas and opinions, often tend to ignore the interests and needs of their employees, even achieve their own goals by devaluing the performance of their subordinates, and hogging their subordinates’ honors and rewards (De Vries and Miller, 1985; Khoo and Burch, 2008; Liu et al., 2017). In the long run, the emotion and dependence between narcissistic leaders and employees will inevitably be seriously damaged, which is not conducive to the cultivation and development of superior-subordinate *guanxi* and will obviously reduce the quality of LMX. At the same time, narcissistic leaders can create an impression of being deceptive, hostile and intimidating through a series of self-promotion techniques (Ouimet, 2010; Back et al., 2013), thus undermining the mutual trust and respect between leaders and subordinates, thereby reducing LMX. In addition, as narcissistic leaders are typical egoists, they will gradually expose their egoistic tendencies in the process of interacting with their subordinates. For example, narcissistic leaders will always treat their subordinates with indifference, deprive their of the opportunities to show their talents, and belittle their performance, which will lead their subordinates to be extremely disappointed with their leaders, thus breeding negative emotions and negatively affecting LMX, causing the subordinates in the circle to jump out of the circle of narcissistic leaders. Therefore, we hypothesize the following:

**Hypothesis 2.** Narcissistic leadership is negatively related to LMX.

In high-quality LMX, employees receive more organizational support and available resources from their leaders, which stimulates a strong sense of responsibility and rewarding behavior, which directly manifests itself in a closer working relationship with the organization (Volmer et al., 2012; Saeed et al., 2019). Specifically, in the context of high-quality LMX, leaders will assign reasonable work tasks to their subordinates and give the subordinates a certain degree of work autonomy, which will make employees feel the trust and support of the leaders, make employees feel that they are in the leader’s circle, and think that they are capable of doing their current work and that they can get help and guidance in time in the case of difficulties. Meanwhile, employees will be more adaptable to their work and give full play to their talents. Thus, the matching degree between individuals and the organization will increase, and the degree of employees’ job embeddedness will naturally increase. Higher levels of LMX indicate that employees are more connected to the organization and that employees are more deeply connected to the organization. Employees who establish high levels of LMX are less likely to leave the organization than those with poor LMX. Xu et al. (2018) showed that high-quality LMX also enhances leaders’ understanding of their employees and better grasps their work dynamics, thus enabling them to be accurately matched to job resources and positions that are appropriate for their personal development and enhancing employee-organizational fit. Conversely, low-quality LMX has the opposite effect on employee-organization connection, fit, and cost of leaving. In summary, narcissistic leaders reduce LMX levels, and low levels of LMX further reduce employee job embeddedness. Therefore, we hypothesize the following:

**Hypothesis 3.** LMX plays a mediating role in the relationship between narcissistic leadership and employees’ job embeddedness.

### The Mediating Role of Perceived Insider Status

According to social exchange theory and organizational socialization strategies, perceived insider status refers to the extent to which individuals perceive themselves as “insiders” in the organization. Perceived insider status influences numerous important organizational outcomes and is an important mediating variable in explaining the relationship between leaders and employees. In dealing with employees, narcissistic leaders tend to act recklessly for their own personal interests, mainly by doing whatever it takes to obtain personal achievements, abusing, denying, and criticizing employees to maintain their own authority and sense of control (Back et al., 2013). This behavior not only weakens employees’ self-esteem and identification with the organization, but also increases their resentment toward their work, rust the distance between them and the organization, and reduce their perception of insider status. At the same time, the over-sensitive characteristics of narcissistic leaders can lead to their distrustful and defensive mentality toward employees, and fear to take the negative consequences and risks caused by the others’ mistakes or incompetence. The narcissistic leaders need to maintain their authority and status through centralization of power. Therefore, under the management of narcissistic leaders, employees tend to listen to the instructions given by the leaders and cannot make their own decisions, play freely, let alone oppose and question the decisions of the leaders and challenge the authority of the leaders. However, the self-determination theory states that individuals have three basic needs: autonomy, competence, and relationship (Wang S. et al., 2021; Xiang et al., 2021). Narcissistic leaders who are overly sensitive rarely authorize and let employees participate in decision-making or free action, which cannot meet their needs for autonomy. In addition, doubting employees’ abilities, rejecting suggestions put forward by employees, and taking less initiative to care about employees also fail to meet employees’ competency and relationship needs. These can reduce employees’ sense of control over their work and make them perceive that they are not recognized and accepted by their leaders and the organization, thus making it difficult for them to perceive themselves as insiders of the organization (Nevicka et al., 2018). Therefore, we hypothesize the following:

**Hypothesis 4.** Narcissistic leadership is negatively related to perceived insider status.

The resource conservation theory points out that employees with a high perception of insider status receive more support, attention, and operational resources from the organization, which has a positive effect on enhancing employees’ need for respect and social needs and motivating them to exhibit pro-organizational behaviors (Hobfoll, 1989, 2011; Hobfoll et al., 2018). Zhang et al. (2020) showed that employees with higher insider status perceptions proactively strengthen ties with their leaders and colleagues and enhance organizational identity, and thus reducing the propensity to leave. On the contrary, employees with a lower perception of insider status lack sufficient organizational resources to establish a strong connection and fit with the organization, and there is no significant emotional or material cost to them for leaving. In summary, narcissistic leaders reduce employees’ insider identity perceptions, and lower insider identity perceptions in turn reduce employees’ job embeddedness. Therefore, we hypothesize the following:

**Hypothesis 5.** Perceived insider status plays a mediating role in the relationship between narcissistic leadership and employees’ job embeddedness.

### The Chain Mediating Role of Leader-Member Exchange and Perceived Insider Status

Combining the above hypotheses, this paper hypothesizes that LMX and perceived insider status may play a chain mediating role in the relationship between narcissistic leadership and employees’ job embeddedness. Narcissistic leaders are usually obsessed with themselves and ignore the psychological needs and developmental needs of their subordinates, and thus a poor LMX will be established between them. According to social exchange theory, it is known that low quality of LMX not only makes employees unable to get care and help emotionally, but also unable to get sufficient resources materially. In this case, employees tend to feel that they are “outsiders,” and thus lowly losing their enthusiasm for their work and gradually disengaging from the organization. Thus, their perception of insider status will be weakened. From the derivation of hypothesis 5, it is clear that the weakening of employees’ perceived insider status in turn reduces their job embeddedness. Therefore, we hypothesize the following:

**Hypothesis 6.** LMX and perceived insider status play a chain mediating role in the relationship between narcissistic leadership and employees’ job embeddedness.

**FIGURE 1:**
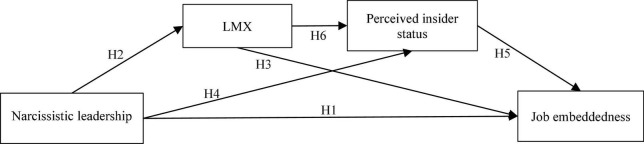
Theoretical model.

Figure 1 shows our theoretical model.

## Materials and Methods

### Sample and Procedure

We aim to explore how narcissistic leadership affects employees’ job embeddedness. Therefore, in the current study, the data were collected by means of online questionnaire research from enterprise new generation employees in Jilin, Beijing, Guangzhou, and Henan. We collected a total of 480 surveys, and after removing invalid surveys (eliminating surveys with missing values above 20%), we obtained a total of 405 valid surveys, with an effective rate of 84.38%. The demographic characteristics of the valid sample suggest that: In terms of gender, 41.7% of participants were male, and 58.3% of participants were female; In terms of age, 25.9% were aged between 18 and 22, 30.5% were aged between 23 and 26, 22.6% were aged between 27 and 30, 21.0% were aged between 31 and 32; In terms of education, 87.6% of participants had a bachelor’s degree or below, 12.3% of participants had a master’s degree; In terms of working time, 19.3% had work for a year or less, 34.3% had worked for 1–3 years, 35.6% had worked for 4–10, 10.9% had work for 10 years above; In terms of time spent with the current leader, 34.6% of participants had spent for a year or less, 37.3% had spent for 1–3 years, 26% had spent for 4–6 years, 2.2% had spent for 10 years or above; In terms of the current position level, 62.2% of participants were general staff, 25.2% were junior management, 11.6% were middle management and 1.0% were senior management.

### Measures

Unless otherwise noted, responses to all items were measured on five-point Likert-type scales, ranging from strongly disagree (1) to strongly agree (5). All variables in this study were measured from well-established scales that are widely used abroad, and all scales have been shown to be valid in Chinese contexts.

#### Narcissistic Leadership

Narcissistic leadership (NL) was assessed using a six-item scale developed by Hochwarter and Thompson (2012). A sample item is “My leader is a very self-centered person.” In this study, the Cronbach’s α score for the scale was 0.906.

#### Leader-Member Exchange

Leader-member exchange (LMX) was assessed using Scandura and Graen (1984)’s six-item scale. A sample item is “My leader understands my job potential.” In this study, the Cronbach’s α score for the scale was 0.857.

#### Perceived Insider Status

Perceived insider status (PIS) is based on a scale developed by Stamper and Masterson (2002) and uses five items. A sample item is “I feel that I fit into the organization.” In this study, the Cronbach’s α score for the scale was 0.881.

#### Job Embeddedness

Job embeddedness (JE) is based on the scale developed by Crossley et al. (2007) and draws on the research of Ng and Feldman (2012) to select five items. A sample item is “It is difficult for me to leave my current job.” In this study, the Cronbach’s α score for the scale was 0.871.

#### Control Variables

In addition, six individual difference variables, including employees’ gender, age, education, working time, current position level, and time spent with the leader, were used as control variables in this study. We controlled them to rule out alternative explanations and to carry out a more reliable test. All the controlled variables were dummy coded. Gender was coded as 1 for participants who were male and 2 for participants who were female. Age was coded as 1 for participants who were aged between 18 and 22 years old, 2 for participants who were aged between 23 and 26 years old, 3 for participants who were aged between 27 and 30 years old, and 4 for participants who were aged between 31 and 32 years old. Education was coded as 1 for participants who had finished a high school education or below, 2 for participants who had an associate’s degree, 3 for participants who had a bachelor’s degree, and 4 for participants who had a postgraduate’s degree. Working years was coded as 1 for participants who had worked for a year or less, 2 for participants who had worked for 1–3 years, 3 for participants who had worked for 4–10 years, and 4 for participants who had worked for 10 years or more. Current position level was coded as 1 for participants who were general staff, 2 for participants who were junior managers, 3 for participants who were middle managers, and 4 for participants who were senior managers. Time spent with the leader was coded as 1 for participants who had spent with their leader for 1–3 years, 2 for participants who had spent with their leader for 4–10 years, 3 for participants who had spent with their leader for 10 years above.

### Data Analysis

First, Cronbach’s α, composite reliability, and confirmatory factor an analyses (CFAs) were conducted to assess the reliability and validity of the key variables. Common method variance (CMV) was also assessed. Second, we used hierarchical regression analysis to examine the hypothesized relationships. Finally, we used the bootstrapping method to test mediation because of its high power (Preacher and Hayes, 2004, 2008).

## Results

### Reliability and Validity

First, before conducting reliability and validity test, we checked CMV because it is a potential issue in the self-reporting approach research. We used Harmon’ one-factor test by including all of the items of the five variables (i.e., narcissistic leadership, LMX, perceived insider status, and job embeddedness) to examine CMV in SPSS 25.0. When the first emerging factor accounted for over 50% of the extracted variables’ variance, common method bias was suggested and CMV would be a problem. The results demonstrated that the first emerging factor accounted for 35.89% of the explained variance, indicating that CMV was not a significant problem in the present study.

Second, we calculated Cronbach’s α and composite reliability of narcissistic leadership, LMX, perceived insider status, and job embeddedness to examine the reliability. As mentioned above, the values of Cronbach’s α and composite reliability were greater than the threshold value of 0.80, demonstrating acceptable reliability.

Finally, we conducted a series of CFAs using Amos 23.0 on the scales including narcissistic leadership, LMX, perceived insider status, and job embeddedness to examine discriminate validity (see Table 1). Results showed that the fit of the five-factor model in which items were loaded on their respective measures was better than any other model (χ^2^/df = 2.946, RMSEA = 0.069, CFI = 0.923, TLI = 0.912, IFI = 0.923, SRMR = 0.062). These results of CFAs provided full support for the discriminate validity of our study instruments.

**TABLE 1:** Results of confirmatory factor analyses.

Models	Variables	χ^2^	df	χ^2^/df	IFI	RMSEA	CFI	TLI	SRMR
Four-factor model	NL, LMX, PIS, JE	598.138	203	2.946	0.923	0.069	0.923	0.912	0.062
Three-factor model	NL, LMX + PIS, JE	914.267	206	4.438	0.862	0.092	0.862	0.845	0.075
Two-factor model	NL, LMX + PIS + JE	1427.976	208	6.865	0.763	0.120	0.762	0.736	0.092
One-factor model	NL + LMX + PIS + JE	2631.383	209	12.590	0.529	0.169	0.527	0.477	0.152

### Descriptive Statistics and Correlations

We calculated the correlations among study variables using SPSS 25.0. As shown in Table 2, narcissistic leadership was negatively related to LMX (*r* = –0.296, *p* < 0.01), negatively related to perceived insider status (*r* = –0.305, *p* < 0.01), and negatively related to job embeddedness (*r* = –0.123, *p* < 0.01). LMX was positively related to perceived insider status (*r* = 0.518, *p* < 0.01) and positively related to job embeddedness (*r* = 0.445, *p* < 0.01). Meanwhile, perceived insider status was positively related to job embeddedness (*r* = 0.353, *p* < 0.01). These results provided preliminary supports for the hypotheses proposed above. We further used hierarchical regression analysis to test the hypotheses.

**TABLE 2:** Results of correlation analysis.

	1	2	3	4	5	6	7	8	9
1.Gender									
2.Age	0.005								
3.Education	0.002	0.068							
4.Working time	0.031	0.034	–0.004						
5.Current position level	0.045	0.097*	0.079	0.131**					
6.Time spent with the leader	0.006	0.099	–0.004	0.088	0.089				
7.NL	0.067	0.110*	–0.037	0.093	0.057	0.122**			
8.LMX	0.033	0.068	0.048	0.071	0.033	0.034	−0.296**		
9.PIS	0.056	0.012	0.087	0.062	–0.029	0.062	−0.305**	0.518**	
10.JE	0.042	0.054	0.096	0.055	0.135**	0.071	−0.123**	0.445**	0.353**

**p < 0.01, *p < 0.05.

### Hypotheses Testing

Research hypotheses were tested using hierarchical regression analysis. The results in Table 3 showed that (1) compared with Model 5, Model 6 showed that narcissistic leadership had a negative impact on job embeddedness (β = –0.126, *p* < 0.01) after the influence of fixed control variables and can additionally explain the job embeddedness variation of up to 1.5% (Δ*R*^2^ = 0.015). The significant term of narcissistic leadership offered full support for Hypothesis 1; (2) compared with Model 1, Model 2 showed that the regression coefficient of narcissistic leadership was significantly negative (β = –0.314, *p* < 0.001), and an additional 9.7% (Δ*R*^2^ = 0.097) of LMX variation was explained. The results offered full support for Hypothesis 2; (3) compared with Model 6, after the influence of fixed control variables and narcissistic leadership, LMX was significantly positive (β = 0.534, *p* < 0.001) and can extra explain 23.5% (Δ*R*^2^ = 0.235) of job embeddedness, and regression coefficient between narcissistic leadership and job embeddedness was still significant (β = –0.142, *p* < 0.001), indicating that LMX played a partial mediating role between narcissistic leadership and job embeddedness. These results provided support for Hypothesis 3; (4) compared with Model 3, Model 4 showed that the regression coefficient of narcissistic leadership was significantly negative (β = –0.310, *p* < 0.001), and an additional 9.4% (Δ*R*^2^ = 0.094) of perceived insider status variation was explained. The results offered full support for Hypothesis 4; (5) model 4, 6, and 8 showed that after the influence of fixed control variables and narcissistic leadership, perceived insider status was significantly positive (β = 0.440, *p* < 0.001) and can extra explain 16.8% (Δ*R*^2^ = 0.168) of job embeddedness, and regression coefficient between narcissistic leadership and job embeddedness was still significant (β = –0.131, *p* < 0.001), indicating that perceived insider status played a partial mediating role between narcissistic leadership and job embeddedness. These results provided support for Hypothesis 5.

**TABLE 3:** Results of hierarchical regression analysis.

Variables	LMX		Perceived insider status		Job embeddedness			
	Model 1	Model 2	Model 3	Model 4	Model 5	Model 6	Model 7	Model 8
Gender	–0.067	–0.074	0.019	0.012	–0.023	–0.026	0.014	–0.031
Age	–0.031	–0.025	–0.027	–0.021	0.160*	0.163**	0.176***	0.172**
Education	–0.020	–0.048	–0.024	–0.051	0.149**	0.138**	0.163***	0.160***
Working time	−0.176*	−0.159*	−0.178*	−0.161*	−0.219**	−0.212**	−0.127*	−0.141*
Current position	0.213***	0.233***	0.135*	0.155**	0.100	0.108*	–0.016	0.040
Time spent with the leader	0.187**	0.181**	0.113	0.108	0.260***	0.258***	0.161**	0.211***
NL		−0.314***		−0.310***		−0.126**	−0.142***	−0.131***
LMX							0.534***	
PIS								0.440***
*R* ^2^	0.078	0.174	0.037	0.131	0.114	0.129	0.365	0.298
Δ*R*^2^	0.078	0.097	0.037	0.094	0.114	0.015	0.235	0.168
*F*	5.593***	11.980***	2.560*	8.570***	8.526***	8.427***	28.432***	20.967***

***p < 0.001, **p < 0.01, *p < 0.05.

To further test the mediation effect of LMX and perceived insider status, we used the procedures proposed by Preacher and Hayes (2004, 2008) and applied bias-corrected bootstrapping method to further examine the mediation effect through the “Process” plugin of SPSS 25.0. This method can produce higher statistical power. The bootstrapping sample size was set to 5,000, the confidence interval was set to 95%, and the results were shown in Table 4.

**TABLE 4:** Results of bootstrapping mediation effect examination.

Paths	Effect	LLCI	ULCI
Narcissistic leadership→LMX→job embeddedness	–0.167	–0.176	–0.062
Narcissistic leadership→perceived insider status→job embeddedness	–0.136	–0.147	–0.053
Narcissistic leadership→LMX→perceived insider status→job embeddedness	–0.069	–0.058	–0.005

**FIGURE 2:**
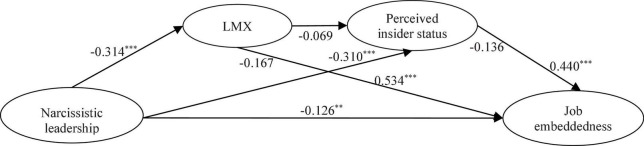
Estimated model. ***p < 0.001, **p < 0.01, *p < 0.05.

The bootstrapping mediation analysis showed that at the 95% confidence interval level, (1) the indirect effect of LMX between narcissistic leadership and job embeddedness was –0.167 and the confidence interval (LLCI = –0.176, ULCI = –0.062) did not included 0, indicating that Hypothesis 3 got full supported. (2) the indirect effect of perceived insider status between narcissistic leadership and job embeddedness was –0.136 and the confidence interval (LLCI = –0.147, ULCI = –0.053) did not include 0, indicating that Hypothesis 5 got full supported. (3) the indirect effect of LMX and perceived insider status between narcissistic leadership and job embeddedness was –0.069 and the confidence interval (LLCI = –0.058, ULCI = –0.005) did not include 0, indicating that Hypothesis 6 got full supported (Cepale et al., 2021). The estimated model was shown in Figure 2.

## Discussion

The current study explores the relationship between narcissistic leadership and employees’ job embeddedness based on social exchange theory and resource conservation theory. The findings show that narcissistic leadership can affect employees’ job embeddedness through three indirect paths: narcissistic leadership affects employees’ job embeddedness by reducing LMX; narcissistic leadership weakens employees’ perceived insider status, which in turn reduces their job embeddedness; narcissistic leadership further reduces job embeddedness by lowering LMX and weakening employees’ perceived insider status.

### Theoretical Implications

First, research on narcissistic leadership in China is mainly at the superficial level, such as its connotation definition, and related empirical studies are relatively narrow in scope. The current study explores the effects of narcissistic leadership on employees’ job embeddedness based on the Chinese context, which not only verifies the mechanisms and effects between narcissistic leadership and employees’ job embeddedness but also enriches the research on related variables. We localizes narcissistic leadership in the Chinese cultural context. Narcissistic leadership is proposed in the western cultural context, and most of the existing empirical studies have been conducted with employees in western countries as the sample. Since China has a different cultural context from the west, where collectivism has a significant role in orienting organizational members, the present study enriches the empirical analysis of narcissistic leadership in the Chinese context, while clarifying the negative effects of narcissistic leadership on job embeddedness and expanding the scope of research on leadership styles affecting employee behavior, as most previous literature has focused on positive leadership style types.

Second, this study uses the social exchange theory and the resource conservation theory as entry points to explore the effect of narcissistic leadership on employees’ job embeddedness, which not only provides a sturdy theoretical basis for the mechanism of narcissistic leadership, but also further enriches the practical application of the theory in the business and improves the persuasive power of the theory. With the help of social exchange theory and resource conservation theory, the current study uncovered two “keys” that link the relationship between narcissistic leadership and job embeddedness, that is, LMX and perceived insider status. On this basis, we developed a research model of narcissistic leadership influencing job embeddedness, revealing the complex influences of LMX and perceived insider status on the relationship between narcissistic leadership and job embeddedness. On the one hand, it can prove that LMX and perceived insider status can independently enhance employees’ job embeddedness. On the other hand, it can verify whether LMX and perceived insider status can play separate mediating role between narcissistic leadership and job embeddedness. Moreover, it provides a research context for integrating social exchange theory and resource conservation theory.

Finally, based on the review of related literature and clarification of the relationship between the variables, the present study constructs the first chain mediation model of “narcissistic leadership → LMX → perceived insider status → job embeddedness,” which opens the “black box” between narcissistic leadership and employees’ job embeddedness and completes the research perspective of the current single mediating variable of narcissistic leadership. In addition, we examines the role played by both LMX and perceived insider status between narcissistic leadership and job embeddedness, and develops a careful examination of their mediating effects, which is an important addition to the existing research.

### Practical Implications

In addition to the theoretical implications, this study also provides guidance for practitioners. On the one hand, narcissistic leadership will reduce LMX and perceived insider status, resulting in large-scale flow of personnel in the organization and huge talent loss to the organization. Therefore, enterprises can take the following measures to ease and restrict the “dark” effect of narcissistic leaders. In the process of selecting leaders, extra care should be taken to screen candidates with high levels of narcissism and prominent negative characteristics. Narcissistic leaders can be arranged in positions that require an innovative spirit and risk-taking to highlight the charismatic strengths of narcissistic leaders, rather than in positions that lead new generations of employees. Matching narcissistic leaders with suitable employees when developing staff matching programs. The current study found that narcissistic leaders cannot promote job embeddedness in new generation employees, so it is necessary to avoid the new generation employees when matching subordinates for narcissistic leaders. Organizations should establish a supervision mechanism and control system to improve the transparency of leadership behavior, improve the reward and punishment system to restrain the undesirable behaviors of narcissistic leaders, guide them to develop positive traits, reduce their excessive self-focus, and make them focus more on improving employees’ embeddedness, stabilize organizational performance, and promote corporate development. In addition, organizations can use the system to confine leaders’ narcissistic tendencies and motivate them to prioritize collective interests over personal self-interest. Organizations can also create a harmonious working atmosphere, enhance employees’ sense of participation and belonging, and promote employees to have positive behaviors conducive to leadership and organization.

On the other hand, since LMX and perceived insider status can enhance new generation employees’ job embeddedness, leaders, especially narcissistic leaders, should properly manage the relationship pattern with new generation employees. Based on the Chinese cultural context, employees attach great importance to relational orientation in the organizational environment. However, due to multiple factors, there are differences in the level of relationship between leaders and different subordinates, which will lead to the fact that the resource allocation of leaders to subordinates is based on the quality of relationship, which can be detrimental to the interests of employees who do not get sufficient resources. To avoid perception of unfair treatment, leaders should try to treat all subordinates in a similar and supportive manner. This cannot only improve the relationship between subordinates and leaders, but also enhance employees’ perception of insider status and increase their connection to the organization.

### Limitations and Future Research

Our study has several limitations. First, the surveys in our study were mainly self-assessed by new generation employees. Although the Harmon’ one-factor test was used to verify that there was no serious common method bias, it was not possible to completely exclude the possibility of its existence. Future research can used a staged data collection approach to weaken common method bias. Second, the scales used in our study were all mature foreign scales, which were obtained by back-translation method, but further validation is needed to determine whether they are suitable for the Chinese context. Third, the cross-sectional data used in this study may limit the interpretation of causal relationships, so longitudinal follow-up of the data could be considered in the future. Finally, although the present study addresses that leaders’ narcissistic behaviors can negatively affect new generation employees’ job embeddedness, we have not discussed whether the narcissistic attributes of the new generation employees can have a negative impact on other employees. If so, what the transmission mechanisms are requires further exploration by future research.

## Data Availability Statement

The original contributions presented in this study are included in the article/supplementary material, further inquiries can be directed to the corresponding author.

## Author Contributions

HW was primarily responsible for designing the study, collecting and analyzing data, and drafting the manuscript. RJ made several revisions and refinements to the content of the manuscript. FL helped collect some of the data and drafted the manuscript. All authors made significant contributions to the study concept and design.

## Conflict of Interest

The authors declare that the research was conducted in the absence of any commercial or financial relationships that could be construed as a potential conflict of interest.

## Publisher’s Note

All claims expressed in this article are solely those of the authors and do not necessarily represent those of their affiliated organizations, or those of the publisher, the editors and the reviewers. Any product that may be evaluated in this article, or claim that may be made by its manufacturer, is not guaranteed or endorsed by the publisher.
